# The Bone—Vasculature Axis: Calcium Supplementation and the Role of Vitamin K

**DOI:** 10.3389/fcvm.2019.00006

**Published:** 2019-02-05

**Authors:** Grzegorz B. Wasilewski, Marc G. Vervloet, Leon J. Schurgers

**Affiliations:** ^1^Department of Biochemistry, Cardiovascular Research Institute Maastricht, Maastricht University, Maastricht, Netherlands; ^2^Nattopharma ASA, Hovik, Norway; ^3^Department of Nephrology and Amsterdam Cardiovascular Sciences, Amsterdam University Medical Centers, Amsterdam, Netherlands

**Keywords:** calcium paradox, vitamin K, vascular calcification, calcium supplements, bone loss

## Abstract

Calcium supplements are broadly prescribed to treat osteoporosis either as monotherapy or together with vitamin D to enhance calcium absorption. It is still unclear whether calcium supplementation significantly contributes to the reduction of bone fragility and fracture risk. Data suggest that supplementing post-menopausal women with high doses of calcium has a detrimental impact on cardiovascular morbidity and mortality. Chronic kidney disease (CKD) patients are prone to vascular calcification in part due to impaired phosphate excretion. Calcium-based phosphate binders further increase risk of vascular calcification progression. In both bone and vascular tissue, vitamin K-dependent processes play an important role in calcium homeostasis and it is tempting to speculate that vitamin K supplementation might protect from the potentially untoward effects of calcium supplementation. This review provides an update on current literature on calcium supplementation among post-menopausal women and CKD patients and discusses underlying molecular mechanisms of vascular calcification. We propose therapeutic strategies with vitamin K2 treatment to prevent or hold progression of vascular calcification as a consequence of excessive calcium intake.

## Introduction

Calcium is an abundant element in nature and is a major component of sedimentary rock that covers 75 to 80% of the Earth's surface. Calcium is also widely abundant in the human body, primarily in bone, and teeth. Calcium salts are occasionally found outside bone in a variety of tissues; this is broadly termed as extra-skeletal calcification. In these extra-skeletal sites, calcium exists in multiple forms, including amorphous calcium phosphate, hydroxyapatite, and magnesium whitlockite. A remarkable observation is that under several pathological conditions, as will be discussed, calcium mineral content of bone declines, while it is increasing on these extra-osseous sites. This has been termed the “calcium paradox” and was introduced to describe the paradoxical correlation between lower bone calcium content with parallel increased vascular calcium content (1). The calcium paradox refers to epidemiological data reporting that postmenopausal women experience bone loss, yet simultaneously screen positive for vascular calcification. This phenomenon is common in osteoporotic women and patients suffering from chronic kidney disease (CKD). Prevalence and morbidity of both cardiovascular disease and osteoporosis are increasing in the global population. Such observations have been noted in several studies, where a correlation of low bone mineral density (BMD) was associated with increased cardiovascular mortality (2–6).

The use of calcium supplements has been widely advised due to their assumed ability to support bone health and BMD (7, 8). Calcium is an essential element for bone growth during childhood (9), as well as in preserving bone mineral density during adolescence (10). However, a systematic review and meta-analysis of the effects of calcium supplementation along with vitamin D treatment showed that this treatment was not associated with a lower incidence of fracture risk in adults, questioning whether calcium supplementation contributes to the maintenance of healthy bone (11). In turn, recent data suggest that calcium supplements increase prevalence of myocardial infarction (12), and may increase risk of coronary artery calcification (CAC) (13). Moreover, higher doses of calcium from supplements than calcium obtained from dietary intake might promote cardiovascular calcification (14). Thus, despite the relative benefit of calcium supplementation for bone, calcium supplements became controversial because of a possibly increased cardiovascular risk. Substantially different from calcium from dietary sources, calcium form supplements induce an acute rise in serum calcium levels that highly oscillates in blood for up to 6 h (15, 16). The plasma calcium concentration is tightly regulated by vitamin D, parathyroid hormone (PTH), and calcitonin (17, 18).

Vitamin K-dependent proteins (VKDP) also play an important role regulating mineralization both in bone and the vasculature. Osteocalcin (OC) is produced exclusively by osteoblasts and supports the binding of calcium to the bone mineral matrix, whereas matrix Gla-protein (MGP) is synthesized by vascular smooth muscle cells and chondrocytes to prevent ectopic calcification. While hepatically produced coagulation factors are the prototypical VKDP, the extra-hepatic VKDP also unequivocally need vitamin K as cofactor to become biologically active. Related to that, vitamin K2 has been shown to prevent bone loss and strength and prevents stiffening of arteries (19, 20). Western diet does not provide sufficient vitamin K to activate all OC and MGP that is produced (21, 22). Also in CKD patients, vitamin K deficiency is prevalent, so K2 supplementation has been suggested as treatment option to attenuate vascular calcification (23, 24).

In this review we provide the latest insights of the calcium paradox and the potential of using vitamin K to support both bone and vascular health.

## Bone Metabolism

Calcification generally is a physiological process, necessary to build bone and dentin. Bone provides structural support, strength, necessary for locomotion, and protection from the environment. The balance in bone formation and bone resorption is crucial for optimal bone health. A disturbed balance of this process results in bone loss and is termed osteoporosis. During childhood bone is formed and bone peak mass is achieved during young adulthood, after which bone mass gradually declines. Bone loss is the consequence of bone resorption outbalancing bone formation (25). This is accompanied by bone architectural changes including trabecular bone becoming thinner, less abundant, and osteoclastic perforation of cortical bone (26).

### Bone Formation

The skeleton is systematically renewed in the process of bone remodeling to maintain strength and rigidity. Bone remodeling can be considered to be part of calcium homeostasis system and enables the skeleton to adapt to changes. Bones adapt their structure depending on their function, mechanical strain and need for stability during development. It is mediated on the surface of cortical and trabecular bone, and at anatomically different sites named basic multicellular subunits (27).

Two pathways of bone formation exist, together termed osteogenesis. The first is known as endochondral ossification and involves a differentiation of mesenchymal cells into chondrocytes or osteoblasts (28, 29). As chondrocytes mature, they expand in size and become hypertrophic and eventually undergo apoptosis, secreting vesicles that initiate mineralization of extracellular matrix (30). As they die, with vascular evasion and matrix remodeling (osteoclast mediated), the calcified cartilage is subsequently replaced by bone. Nestin-positive mesenchymal progenitors associated with the invading vasculature differentiate into bone-forming osteoblasts and deposit a type I collagen-based bone matrix on the degraded cartilage template (31), (32). The second process of bone formation is intramembranous ossification. First, mesenchymal cells directly differentiate into osteoblasts, which are bone-forming cells. Next, type I collagen matrix is deposited by these cells, that can bind calcium salts, which form hydroxyapatite crystals. This mineralization of the matrix underlies the strength and compactness of the bone. With time, osteoblasts eventually become trapped in calcified extracellular matrix and transdifferentiate into osteocytes. Osteoblasts are the only bone cell type releasing the vitamin K-dependent protein OC (discussed below). As the newly formed bone is laid, its deposition must be tightly regulated to maintain homeostasis. This balance is achieved by bone-resorbing cells, entering the blood vessels of bone, which are termed osteoclasts and are of macrophage origin. Each osteoclast is able to secrete hydrogen ions, thereby acidifying the bone surface dissolving mineralized matrix, followed by interactions that enhance the action of osteoblasts (33–35). Upon resorption, bone-matrix embedded osteocalcin is released contributing to its circulating levels (36).

### Bone Loss

Bone loss is most typical in women after reaching the age of 50 years following menopause. The pattern of sex hormonal secretion drastically changes after the menopause, resulting in disbalance in bone turnover markers, making postmenopausal women susceptible to osteoporosis and fractures. Remarkably cardiovascular diseases are also more prevalent in postmenopausal women. Therefore, it is important to understand the molecular mechanisms by which hormonal changes leads to both osteoporosis and cardiovascular disease (37, 38). The post-menopausal period is accompanied by substantial reduction of estrogen levels leading to bone resorption, yet simultaneously reducing calcium absorption (39). It is not the aim of this review to elaborate on the effect of estrogen on the vasculature [reviewed elsewhere (39)]. Instead, we will focus on specific pathways involved in calcium metabolism.

PTH is released upon hypocalcemia, indirectly stimulating release of calcium from bone. In CKD, autonomous production of PTH may occur. Additionally, PTH promotes reabsorption of ultra-filtered calcium in distal tubules and activates vitamin D thereby increasing circulating calcium levels by raising gastrointestinal uptake of calcium (17, 18). Calcium-sensing receptors (CaR) present on the surface of parathyroid glands enable sensing of circulating calcium concentration (40), contributing to calcium modulation.

Vitamin D is a fat-soluble vitamin that can be obtained from diet, sun exposure, or supplements, and is metabolized by a series of enzymatic reactions in the body producing its active 1,25-dihydroxyvitamin D form (41, 42). Vitamin D (in inactive form) is often prescribed in combination with calcium supplements. Active 1,25-dihydroxyvitamin D enhances absorption of intestinal calcium and phosphate thus contributing to the regulating of mineral balance (43, 44). In the absence of vitamin D, only 10–15% of intestinal calcium is absorbed, which can be increased to 30–40% in the presence of active vitamin D (45, 46). Vitamin D was found to stimulate production of vitamin K-dependent proteins, like osteocalcin (47). Osteocalcin is a protein involved in bone mineralization [reviewed elsewhere (48)]. Remarkably, inclusion of vitamin K in calcium and vitamin D supplements improved BMD and ucOC when compared with vitamin D and calcium alone (49).

CKD patients often experience deficiency of 1,25-dihydroxyvitamin D as a consequence of lost kidney mass and the effects of fibroblast growth factor 23 (50), resulting in declined activity of 1-alpha hydroxylase (51–53). Reduced serum levels of 1,25-dihydroxyvitamin D lead to hypocalcemia on top of positive phosphate balance, both stimulating PTH release and eventually leading to secondary hyperparathyroidism.

## Vascular Calcification

Vascular calcification is a pathological process, and has been firmly established as a risk factor for cardiovascular events and mortality (54, 55). Vascular calcification is a process of extraosseous mineral deposition in blood vessels, including large arteries such as aorta, carotid arteries, iliac arteries, and cardiac valves. Bone mineralization and vascular calcification share many similarities, including expression of bone-related proteins in the vasculature and secretion of extracellular vesicles (EVs) both preceding the phase of calcification (56, 57). Vascular calcification can occur either in the tunica media or tunica intima of the vessel wall. Medial calcification is also known as Möckenberg's sclerosis and involves vascular smooth muscle cell (SMC) calcification in the absence of previous local lipid accumulation, and inflammation. Medial calcification is related to CKD, diabetes mellitus, and aging, and results in increased arterial stiffness and risk of cardiovascular events (58, 59). In contrast, intimal calcification is associated with atherosclerotic plaque formation and the amount of calcification is considered to be a measure of atherosclerotic burden (1).

For many years vascular calcification was considered as a clinically irrelevant process reliant of passive deposition of calcium crystals, merely reflecting a passive feature of disease and aging. Recent evidence however suggests otherwise, and vascular calcification appears to be a highly regulated process. SMCs release calcification inhibitors, thus efficiently preventing spontaneous calcification in spite of supersaturation of extracellular calcium and phosphate levels (60).

### Vascular Smooth Muscle Cell Phenotypic Switching

SMCs are the main cellular component of the tunica media in arterial vessels providing structural support and regulating vascular tone and elasticity to alterations in pressure conditions. In physiology SMCs possess a contractile phenotype and express contractile-specific markers, including alpha-smooth muscle actin, calponin, and SM22alpha, enabling them to perform contraction of the vessel wall [reviewed elsewhere (61, 62)]. SMC function is associated with a high level of phenotypic plasticity in order to perform a variety of functions including production of extracellular matrix and repair (61, 63). Several factors have been implicated in regulating SMC phenotype, including mineral imbalance (calcium, magnesium, and phosphate-induced loss of calcification inhibitors and presence of calcification promotors) (64). Downregulation of contractile markers is a hallmark for SMC phenotypic switching (65). It has been shown that phosphate can induce an osteochondrogenic phenotypic switching of SMC, as will be outlined in more detail below (61, 66–69), whereas elevated calcium levels shift the contractile phenotype toward a synthetic SMC phenotype (57). Both calcium- and phosphate- induced phenotypic switching are associated with an increase in the secretion of calcifying extracellular vesicles (56, 57).

### Elevated Phosphate Levels Promote Osteochondrogenic Differentiation of SMCs

CKD patients often develop medial calcification (70). In CKD, a strong correlation between serum phosphate levels and vascular calcification is present (71, 72). In an animal model of CKD, arterial calcification developed after feeding animals a phosphorous-rich diet only (73). Initiation and progression of calcification in CKD patients correlates with impaired mineral metabolism represented by elevated serum level of phosphate and/or calcium (74). Moreover, high circulating phosphate levels have been linked to increased cardiovascular morbidity even among young people receiving dialysis (75) and in CKD patients (76). *In vitro*, elevated phosphate levels result in upregulation of bone-like markers in SMC including osterix, alkaline phosphatase (ALP), and Runx2, and downregulation of SMC contractility markers (77). SMC cultured in osteogenic cell culture media differentiate into calcifying-SMC resembling osteoblasts (68). In aortic valves of patients with aortic stenosis, valvular interstitial cells demonstrate similarities with osteoblasts (78), which also exhibit lamellar bone formation (79). Upon injury or in atherosclerosis, SMCs induce the release of platelet-derived growth factor (PDGF) similarly to platelets (80, 81). SMC are known to express the PDGF receptor subtypes and the level of expression is greatly increased in connective tissue and in SMCs followed by PDGF stimulation (82).

## The Calcium Paradox

The paradoxical co-existence of declined calcium-mineral content in bone, and parallel increased arterial calcification, as a consequence of impaired calcium metabolism, is termed the calcium paradox. This is most pronounced in post-menopausal women and CKD patients. Many studies have consistently shown a coexistence of osteoporosis in post-menopausal women and increased calcification of either abdominal aorta and carotid arteries (5, 83–90). Such paradox of decreased bone mineral density and vascular calcification has also been documented in a population study of middle-aged men, suggesting it is not unique to women (91), and pointing to a specific metabolic abnormality. In patients with CKD disturbed calcium and phosphate homeostasis is present and many studies consistently reported bone abnormalities including decreased BMD and fractures and coexistence of increased vascular calcification and all-cause mortality (92–108).

Kidney Disease: Improving Global Outcomes (KDIGO) guidelines recommend the term chronic kidney disease-mineral bone disorder (CKD-MBD) to express this clinical syndrome encompassing mineral (e.g., calcium), bone, and cardiovascular calcification abnormalities that develop as a complication of CKD (109). In addition to bone disease, patients with CKD are also prone to vascular calcification, bone fragility and fractures. It has been shown that patients on dialysis, which is the end stage of CKD (CKD stage 5D), have an increased risk of fractures (110, 111) and vascular calcification (112), and therefore the calcium paradox also exists in CKD patients. CKD pathological characteristics include biochemical imbalances leading to elevated levels of circulating phosphate (113–115). In untreated patients, circulating calcium levels are decreased due to vitamin D deficiency, whereas vitamin D supplementation might be beneficial in improving biochemical endpoints in CKD patients (116). Vitamin D is often used in combination with calcium supplementation therapy. In patients on dialysis, coronary artery calcification is prominent and contributes to high mortality and morbidity. However, this use of both calcium and vitamin D, while being possibly protective for bone disease, may aggravate vascular calcification. Uraemia-related cardiovascular risk factors, including hyperphosphatemia and elevated Ca x P product, correlate with quicker onset of vascular calcification (117). Circumventing this calcium paradox may be accomplished by VKDP (118, 119), as will be outlined below.

## Agents That Alter Tissue Mineralization

In the following sections we will discuss treatments known to influence bone and vascular mineralization, and how they might impact calcium metabolism.

### Calcium Supplements

Calcium is important for optimal bone health throughout life. Although dietary intake of calcium may suffice to meet the recommended daily intake, calcium supplements may be an option if diet falls short. dose Globally, recommendations for daily calcium intake vary. The Institute of Medicine (IOM) recommends a daily intake of 1,000 mg/day for men aged 19–70 years and women 19–50 years old, and 1,200 mg/day for older individuals (92) whereas National Osteoporosis Society suggests an intake between 800–1,000mg a day (120). While calcium intake comes from dietary sources such as dairy products, certain vegetables, and fortified foods, many people do not achieve the recommended intake from diet alone. It is estimated that ~35% of the adult U.S. population uses calcium supplements (121). Calcium plays a vital role in various physiological activities, such as nerve conduction, muscle contraction, blood clotting, protein folding, brain function, and regulated cell death (apoptosis) (122, 123). Such broad function of calcium in the body requires precise regulation, and calcium oscillates between 2.15 and 2.60 mmol/L for total plasma calcium in adults and between 1.17 and 1.33 mmol/L for plasma ionized calcium as free calcium represents some 45% of total circulating calcium levels. This free form is the regulated calcium and accounts for bone mineralization as well as pathological calcification (124).

#### Calcium Forms, Absorption, and Effects

Several formulations of calcium are available on the market, differing in bioavailability, and elemental calcium content. Calcium carbonate is the most common form available. However, many studies showed superiority of calcium citrate over calcium carbonate, due to higher bioavailability and because it does not require acidic stomach conditions before ingestion (102). In a study carried out in post-menopausal women supplemented with di-calcium phosphate over a period of 12 months, serum calcium levels did not vary significantly, and only urinary calcium increased progressively in time when compared to the control group. The increased excretion of calcium may indirectly reflect the rise of the renal threshold for excretion and together with the amount of absorbed calcium it may contribute to complications such as deposition in the vasculature (103).

One of the most applied therapeutic intervention for fracture risk is calcium in the form of pills or organic powder. Commercially available calcium is often marketed in combination with vitamin D3 to increase intestinal absorption of calcium (Table 1). It has been proposed that no more than 500 mg of elemental calcium should be taken as single dose to maximize absorption and to avoid side effects, like gastrointestinal complaints (94). When calcium supplements are not exceeding the nutritional daily intake of 800 mg, a low cardiovascular risk was observed (104). Clinical guidelines consider a cumulative calcium intake from foods and supplements that does not exceed 2,000 to 2,500 mg/d, as defined by National Academy of Medicine, as safe for cardiovascular disease outcome (105, 106).

**Table 1:** Comparison of calcium salts frequently used in calcium supplements.

**Calcium salt**	**Elemental calcium % (w/v)**	**Bioavailability**	**Advantages/disadvantages**
Carbonate	40	High (comparable with citrate)	Requires acidic stomach conditions before absorption, might cause acidic rebound, cheap provides greatest amount of elemental calcium
Tricalcium phosphate	38	Moderate (found lower absorption than citrate when used in fortified juice)	High calcium content
Citrate	21	High (higher than lactate/tricalcium phosphate)	Not dependent on stomach acidity, many tablets needed
Gluconate	9	High (comparable with calcium carbonate)	Many tablets needed
Lactate	13	High (comparable with calcium carbonate)	Many tablets needed
Acetate	25	High (scarce information on human subjects)	Inexpensive, wide range of intestine pH absorption
Chloride	27	High (intravenous injection for treatment of hypocalcemia)	Not commonly prescribed low amount of elemental calcium
References		(93, 94, 125)	(11, 33–39, 41)

*Salts are listed according to elemental calcium content which does not necessarily reflect on bioavailability. Absorption is also influenced by stomach acid due to the salt structure e.g., calcium carbonate is basic and needs hydrochloric acid in stomach to produce calcium chloride which is further absorbed*.

Numerous studies and extensive meta-analyses reported on the efficacy and cost effectiveness of calcium supplementation (with or without vitamin D), in improving bone mineral density as well as decreasing fracture risk (83–85, 107, 108). Furthermore, in individuals with inadequate calcium intake, the supplementation plan seems to be beneficial in reducing fragility fractures especially in osteoporotic women (86, 107). Calcium supplementation was also demonstrated to be effective in preventing reduction in bone loss and turnover in healthy population (87). A recent double-blind controlled trial also proved the effectiveness of medium and high calcium intake in maximizing bone mineral density in adolescent girls (88). In addition, many studies described neutral or protective effects of calcium rich foods on cardiovascular outcomes including atherosclerosis, risk of infarction, stroke, and cardiovascular mortality (89, 90, 126–130).

However, recent data challenge the assumption that calcium supplementation improves bone mineral density. A meta-analysis on the correlation between calcium supplementation alone or with vitamin D and bone mineral density in people over 50 years of age demonstrated little beneficial effect (1–2%) in the first year with nearly no further benefits after 1 year on bone mineral density (8). With such low effects it would be challenging to implement calcium supplementation into standard treatment for reduction of fracture risk in the healthy elderly population (131). A recent review summarizing the use and efficacy of calcium supplementation in treating osteoporosis and fracture risk questions the use of calcium supplements because of the weak beneficiary effect on fracture risk while increasing the risk on gastrointestinal problems, kidney stones, and cardiovascular risk (132).

Despite positive outcomes of calcium supplementation, a risk for cardiovascular risk events may exist in specific population. It was recently shown that women who receive calcium supplementation were at higher risk for increased vascular morbidity and mortality, including myocardial infarction (108, 133–139). In turn, recent systematic reviews and meta analyses do not confirm that supplementing calcium (with or without vitamin D) increased prevalence of coronary heart disease, cardiovascular mortality or all-cause mortality, data on which the above-mentioned statement by the National Academy of Medicine is based upon (105, 131). Rapidly elevated transient calcium levels in blood caused by excessive supplementary calcium have been suggested to promote coagulation when compared with placebo in postmenopausal women, likely due to interaction with platelets expressing calcium-sensing receptor (CaSR) (140, 141). Hypercoagulability is considered to have a reinforcing effect on atherosclerosis in animal studies, contributing to cardiovascular disease. Also many coagulation proteins have been described in human atherosclerotic plaques (142). These findings are in line with the association between high calcium intake and cardiovascular calcification in CKD patients (143). Reconciling these sometimes opposing details difficult. There appears to be some protection from fracture risks by calcium supplements, but its safety is still not sufficiently established. Therefore, additional research is still needed.

Calcium-based phosphate binders have been used extensively as a first-choice option since 1970 to alleviate hyperphosphatemia associated with CKD patients due to its low cost, availability, and effectiveness. These calcium-containing phosphate binders are given to CKD patients to complex dietary phosphate, thereby reducing phosphate uptake (144, 145).

As with most supplements, also calcium-containing phosphate binders have side effects, which include abdominal cramps, intestinal bloating, and diarrhea (146). Further, excessive intake of calcium supplements might also result in milk-alkali syndrome and hypercalcemia (92). In addition, also in patients with CKD, the use of calcium-containing binders are associated with progression of CKD, and the recently updated KDIGO guideline suggest to restrict its use (109).

### Vitamin K and Vitamin K-Dependent Proteins

**Figure 1:**
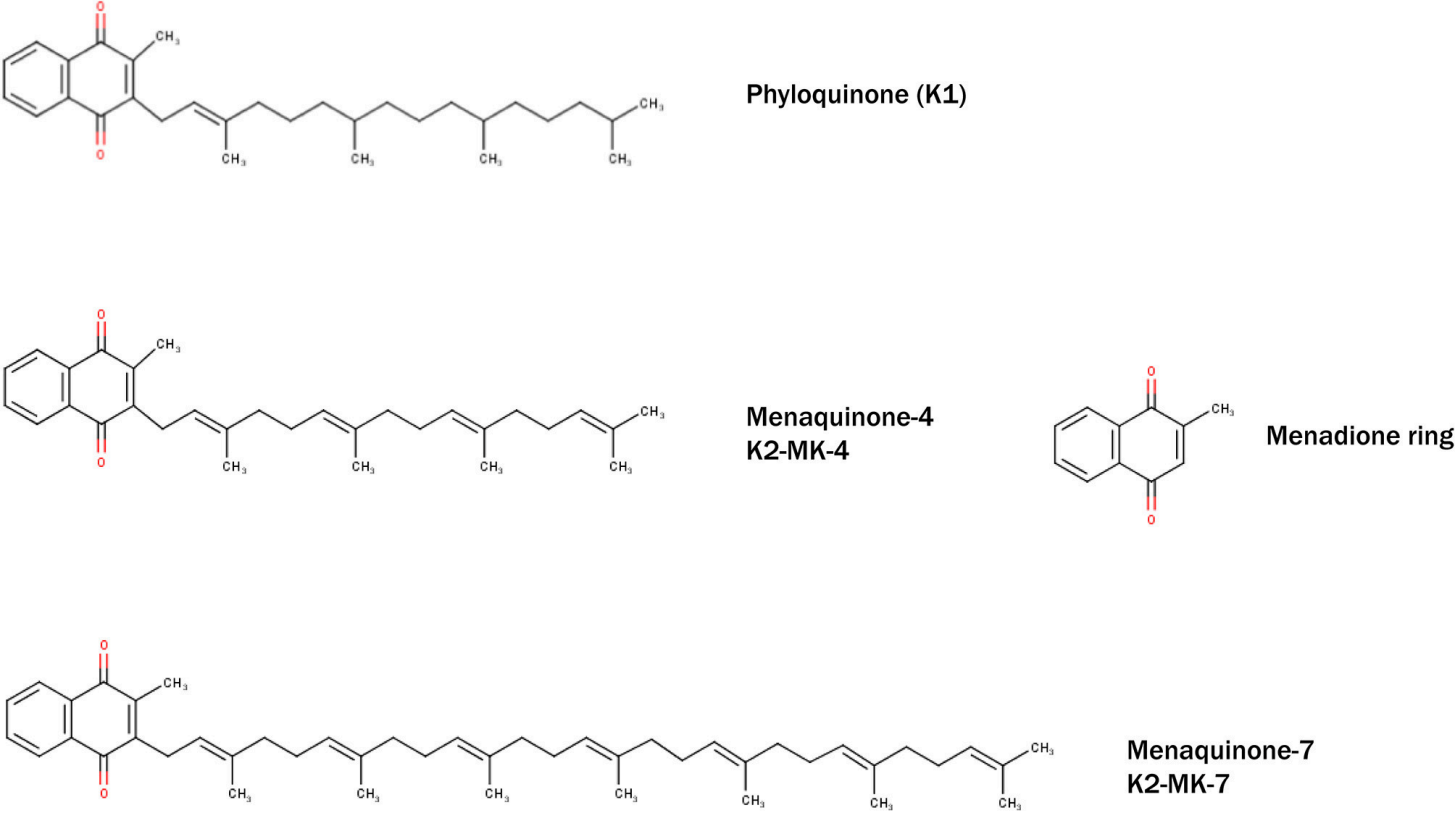
Structural formulae of naturally occurring and biologically active Vitamin K–phylloquinone (K1) and menaquinones (K2-MK-4 and K2-MK-7). All vitamins share common menadione ring (also known as vitamin K3).

Vitamin K was discovered in 1929 by the Danish biochemist Henrik Dam during his experiments on cholesterol metabolism in chickens. When fed low-fat diets, chickens experienced prolonged clotting time and hemorrhage, which surprisingly could not be rescued when diet was enriched with cholesterol. Dam assumed a deficiency of a vitamin required for coagulation, which he termed “Koagulation vitamin,” hence vitamin K (147). Indeed, vitamin K was shown to be a fat-soluble vitamin, consisting a group of structurally related compounds including vitamin K1 (phylloquinone) and vitamin K2 (menaquinones) (Figure 1). Vitamin K1 contains a phytyl chain, whereas K2 is classified according to the length of isoprenoids and indicated as MK-n, where n represents the number of residues. Both vitamins share a common 2-methyl-1,4-naphthoquinone ring, also known as menadione. The main source of vitamin K1 is green vegetables (148), whereas vitamin K2 can be found in fermented foods such as soy beans, cheese, and sauerkraut. The richest source of vitamin K2 (MK-7) is a Japanese dish named *Natto*, which is produced from fermented soy beans with aid of the *Bacillus Subtilis* bacteria strain (149). In addition to nutritional consumption, gut bacteria *Lactococcus* (150) and *Escherischia coli* (151) are able to synthesize long chain menaquinones (Figure 1).

The primary biological function of both K-vitamins is being an unequivocal cofactor in the post-translational modification of VKDP via carboxylation of glutamic acid residues (Glu) to y-carboxylated-glutamic acid residues (152). To fulfill this function, vitamin K needs to be reduced to its active cofactor form (KH2) by quinone reductases. The enzyme y-glutamylcarboxylase (GGCX) oxidizes KH2 to vitamin K-epoxide (KO) (153). Both vitamins K1 and K2 can partake in the activation of VKDP; however, long-chain menaquinones, which are more hydrophobic, have a higher bioavailability and longer half-life and thus bioactivity (154, 155).

VKDP are a group of proteins that require carboxylation of specific protein-bound glutamate-residues, allowing them to bind with high affinity to calcium. This was first demonstrated in coagulation, showing that VKDP of the coagulation cascade need carboxylation to acquire biological activity. This role of vitamin K on coagulation is clinically widely applied by the use of warfarin as anticoagulant treatment. The extra negative charge in VKDP bind via calcium to negatively charged phospholipids to exert their function. In the last three decades, extra-hepatic VKDP have been discovered, including OC, MGP, and Gla-rich protein (GRP; also termed Upper zone of growth plate and Cartilage Matrix Associated protein, Ucma) (156). The function of non-hepatic VKDP has recently be discovered and include prevention of vascular calcification (157) and importantly also promotion of bone metabolism (158). The current knowledge of vascular calcification inhibitors has gained attention of both scientists and clinicians to research their molecular action, aiming to alleviate disease caused by vascular calcification.

#### Osteocalcin

**Figure 2:**
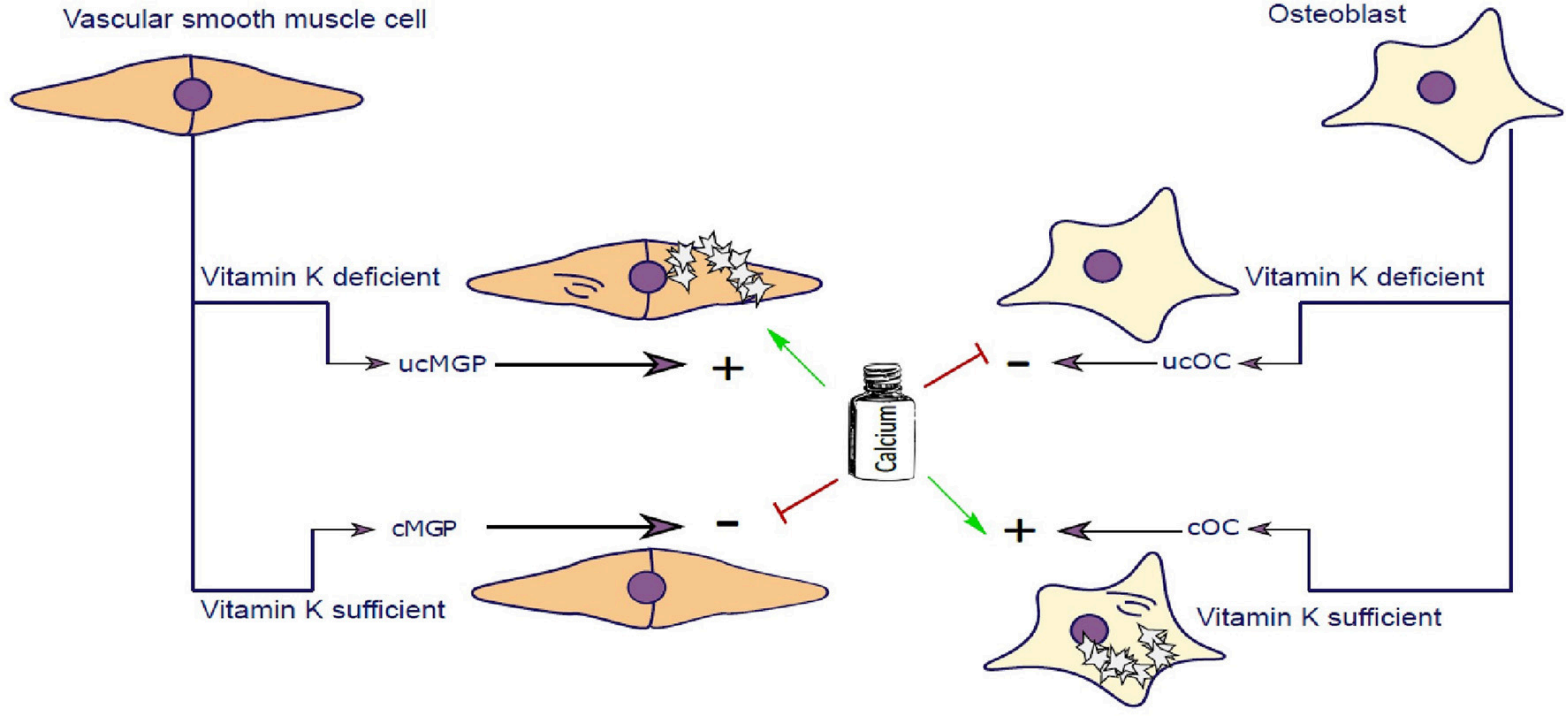
Vascular smooth muscle cells (VSCMC) and osteoblasts are able to synthesize Matrix-Gla-Protein (MGP) and Osteocalcin (OC), respectively. In the presence of vitamin K both proteins are carboxylated (cMGP and cOC) preventing calcification of VSMC and promoting mineralization of Osteoblasts. Vitamin K–dependent carboxylation mechanism keeps extracellular matrix of VSMC free of calcification and simultaneously promotes mineralization of osteoblast matrix. In Chronic Kidney Disease patients, calcium serum levels are elevated further potentiating the calcification of SMCs. Similarly, in post-menopausal women, calcium homeostasis is further impaired contributing to impairment of calcium utilization by osteoblasts. In the event of vitamin K deficiency, both MGP and Osteocalcin are not carboxylated and cannot perform their molecular function.

OC is a major non-collagenous protein abundantly present in bone, responsible for management of skeletal mineralization (159, 160). OC knock-out/null rodents undergo increased bone mineralization, followed by an increase in trabecular thickness, density and bone volume (161–163). During skeletal development, bone mass increases due to the dominant function of osteoblasts which secrete OC, amongst other proteins, enabling bone to grow. In addition to bone function, OC is implicated in stimulating testosterone synthesis and insulin release (164, 165). Other roles of OC are not covered in this review and have been reviewed elsewhere (166). To execute its physiological function, OC needs to be activated by carboxylation, catalyzed by vitamin K. Carboxylated OC (cOC) has a high affinity for calcium ions and aids in forming a hydroxyapatite lattice preceding mineralization of bone (167, 168) (Figure 2). Upon bone degradation, OC, incorporated into mineralized bone, is liberated. Serum OC levels were negatively correlated with bone mineral density (BMD) in post-menopausal woman and healthy subjects (169–171). In a study of healthy girls, plasma phylloquinone was inversely correlated with circulating OC concentrations showing that a better vitamin K status was associated with decreased bone turnover in healthy girls (172).

#### Matrix Gla Protein

The discovery of MGP dates back to 1983 where it was first purified from bovine bone matrix and named after the presence of gamma-carboxyglutamate residues on MGP (173). Shortly thereafter MGP was confirmed to be present in cartilage, lung heart, kidney, and vasculature, with highest protein expression in SMCs and chondrocytes (174–176). Knocking out MGP in mice induced advanced medial calcification and subsequent vessel rupture followed by death in the majority of mice within 6 weeks after birth. This animal model resembles the human Keutel syndrome which is caused by a mutation in the MGP gene (177, 178), which impairs carboxylation of MGP thereby inducing intimal and medial calcification (179). MGP is also dependent on carboxylation of gla-residues, catalyzed by vitamin K, to execute its function as an inhibitor of vascular calcification (Figure 2) (180, 181). Uncarboxylated MGP (ucMGP) is associated with increased risk of vascular calcification, and therefore some researchers advocate that vitamin K status in CKD patients should be carefully monitored (182).

Another mode of action of MGP, besides being an inhibitor of arterial calcification, is inhibition of bone morphogenic protein2/4 (BMP2/4) (183, 184). BMP2 was found to be present in human atherosclerotic lesions (185), acting as downstream signal for osteogenic phenotype switching of SMC by increasing the influx of phosphate into cells (186). In MGP-deficient SMCs, upregulation of osteogenic-specific proteins was notified (187) and it can be speculated that MGP prevents osteogenic transition of SMC by interacting with BMP-2 (188).

#### Gla Rich Protein

GRP, also known as Ucma, is a vitamin K-dependent protein secreted by chondrocytes (189, 190) and present in cartilage, bone (191), and vasculature (192, 193). Despite the creation of GRP knockout mice its precise molecular action remains to be elucidated, because these animals had no manifest deficits in cartilage or bone development (194). So far, the role of GRP has been implicated in calcium regulation in extracellular matrix (156, 192), and thus being an inhibitor of ectopic calcification (192, 193). Indeed, GRP inhibits calcification of aortic tissue by promoting a contractile SMC phenotype via increasing expression of α-smooth muscle actin (193). Moreover, GRP was found to be directly associated with calcium-phosphate crystals suggesting that this protein-crystal interaction modulates calcification (156). Also, in CKD stage 5D, GRP inhibits EV and calcifying protein particles (CPP) induced vascular calcification (195). In addition, GRP was found to promote osteoblast (196) and chondrocyte differentiation (189, 190). More recently, it was shown that GRP inhibited phosphate-induced SMC calcification via BMP-dependent signaling suggesting its role in regulating osteochondrogenic differentiation of SMCs (69). As mentioned above, MGP also inhibits calcification via a BMP-dependent mechanisms (57, 197) and this novel function of GRP function via a BMP-dependent mechanism suggests that both MGP and GRP deficiency contribute to phosphate-induced vascular calcification and cardiovascular risk. Table 2 summarizes vitamin-K dependent proteins involved in calcification.

**Table 2:** Occurrence of selected vitamin K dependent proteins in different tissue compartments.

	**Bone**	**Vasculature**	**Cartilage**
MGP	✓	✓	✓
Gla rich protein (UCMA)	✓	✓	✓
Osteocalcin	✓	?	?
Reference	(173, 191, 198, 199)	(192, 193, 197)	(189–191, 200, 201)

### Phosphate Binders and Vitamin K

**Figure 3:**
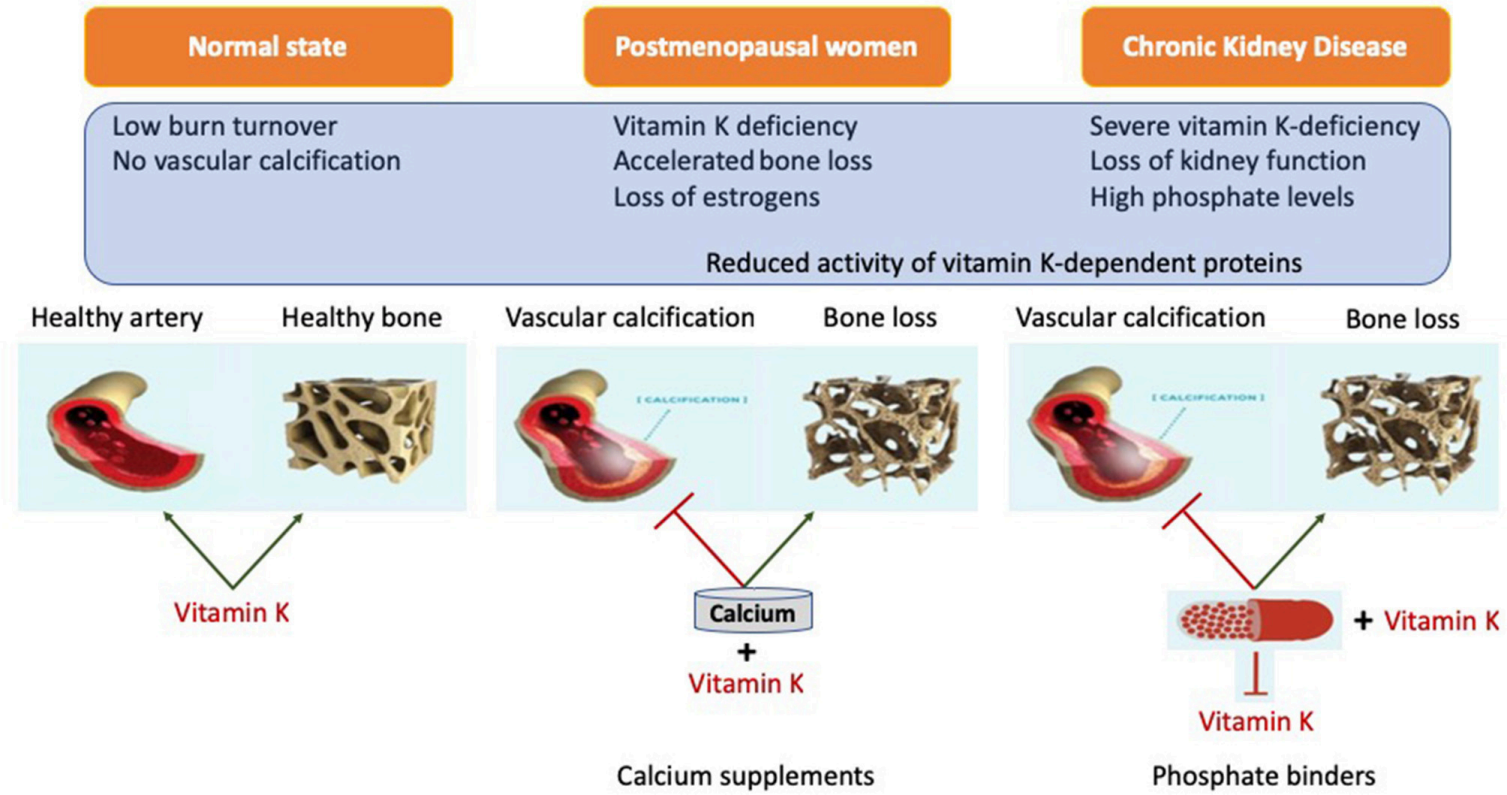
Representation of systemic action of vitamin K on bone and vasculature in the calcium presence. Calcium based phosphate binders are known to reduce the levels of adsorbed phosphate by directly coupling reaction in the gastro-intestinal tract. Phosphate binders were also shown to bind Vitamin K suggesting it might affect its free circulating form. When coupled with phosphate binders, vitamin K is unable to perform its biological function of positively utilizing calcium into the bone and simultaneously acting as calcification inhibitor.

Despite many years of research there is no definite proof that phosphate binders improve outcome, despite their capacity to control phosphate. Although direct studies suggest superiority of non-calcium containing binders over calcium containing binders, it is still unclear if this is due to an advantage of non-calcium containing binders or added risks from calcium containing binders (143, 202–204) Even more striking is that the use of any phosphate binders in earlier CKD, despite lowering phosphate, did not reduce progression of coronary calcification (71). This conundrum may be explained by the recently demonstrated ability of phosphate binders to also bind vitamin K (Table 3). The advantage of lowering phosphate concentrations if thus offset by aggravation vitamin K deficiency. The lack of difference in this CKD patient subgroup could be explained by effective inherent protection in these patients or by simultaneous undesired binding of vitamin K by some phosphate binders resulting in vitamin K deficiency which serves as co-factor for enzymes that activate calcification inhibitors (218, 219) (Figure 3). More recently, it was shown that CKD patients on dialysis treated with the phosphate binder sevelamer revealed higher circulating levels of dp-ucMGP, the inactive form of MGP (221). These findings support the *in vitro* notion and hypothesis that phosphate binders induce a vitamin K-deficiency. Besides the above-mentioned phosphate binders, new forms have recently been developed such as iron-based phosphate binders. Iron oxyhydroxide have been proven to be as potent as sevelamer in decreasing phosphatemia (222), while apparently not interfering with vitamin K-metabolism (218).

**Table 3:** Summary of selected features and effects of available phosphate binders.

**Phosphate lowering agent**	**Binding mechanism**	**Generic name**	**Calcium based**	**Effect on phosphate**	**Effect on Ca x P product**	**Effect on calcium/ hypercalcemia**	**Interaction with vitamin K**
Calcium acetate/magnesium carbonate	Ionic		Yes	↓	↓	↑	Yes
Calcium acetate	Ionic		Yes	↓	↓	↑	?
Calcium carbonate	Ionic	CaCO	Yes	↓	↓	↑	Yes
Lanthanum carbonate	Forms insoluble phosphate complexes	LanCO	No	↓	↓	↓	Yes
Aluminum hydroxide	Ionic	Al salts	No	↓	?	↑	?
Sucroferric oxyhydroxide	Covalent binding	FeSa	No	↓	↓	NS change	No
Sevelamer hydrochloride	Ionic	Sevelamer HCl	No	↓	↓	↓	?
Sevelamer carbonate	Ionic	Sevelamer CO_3_	No	↓	↓	NS change	No
Colestilan	Ionic		No	↓	↓	NS change	?
Bixalomer	?		No	NS change	NS change	NS change	?
Nicotinamide	inhibition of sodium/phosphorus co-transporter	Vitamin B3	No	↓	↓	NS change	?
Ferric citrate	Ionic		No	↓	NS change	NS change	?
Reference	(205, 206)			(207–217)			(218–220)

*Ca, Calcium; P, Phosphorous*.

### Vitamin K to Escape the Calcium Paradox

As outlined, vitamin K has a role in healthy bone formation, while at the same time it provides protection against ectopic calcification, especially in the cardiovascular system. Therefore, it is tempting to speculate that the calcium paradox in fact reflects vitamin K deficiency.

It has been shown that patients with CKD frequently are vitamin K-deficient, which is likely attributable to dietary advice to limit their potassium intake (i.e., intake of green leafy vegetables and thus vitamin K1) and to lower phosphate intake (i.e., intake of dairy products and thus vitamin K2). Besides, these dietary restrictions, especially patients on dialysis frequently suffer loss of appetite, further affecting the intake of essential nutrients, including vitamin K. Another risk for vitamin K deficiency is the use of phosphate binders as outlined above. Finally, use of anticoagulant therapy with vitamin K-antagonists in CKD patients will propel this deficiency even further (223). Although novel direct oral anticoagulants are available, these are often considered unsuitable for patients with a glomerular filtration rate below 30 ml/min/1.73m^2^. Also, in healthy subjects it was shown that the majority has subclinical vitamin K deficiency as deduced from the presence of increased concentrations of uncarboxylated VKDP in the circulation (22, 180, 224). Recent evidence, as outlined in detail above, suggests that vitamin K is an important factor in bone and vasculature in CKD patients and post-menopausal women, and that its role may be overlooked. It creates a window of opportunity to supplement vitamin K in the abovementioned subgroups including CKD patients and post/peri menopausal women frequently deficient in vitamin K.

Although supplementation with vitamin K2 (MK-4) daily for 3 years did not improve bone mineral content or bone mineral density, it did maintain bone strength at femoral neck site in post-menopausal women (19), thus indicating a beneficial effect on post-menopausal bone strength loss. Aside from MK-4's known function for gamma carboxylation, and thereby preventing ectopic calcification to occur, it was shown to also promote maturation of osteoblasts (225) and to suppress osteoclast maturation while promoting their apoptosis (226, 227). MK-7, a long-chain menaquinone, was found to have more beneficial effect on bone and facilitates bone mineralization, including cortical bone structure as compared to MK-4 (228). In support to *in vivo* evidence, several trials assessed the feasibility of MK-7 as treatment for CKD and post-menopausal osteoporotic patients. It was shown that MK-7 (MenaQ7) improves bone strength at the femoral neck via increasing bone mineral content (BMC) and bone mineral density (BMD) (19, 229, 230). In addition, hemodialysis patients supplemented with MK-7 showed a substantial decrease in dp-ucMGP along with ucOC and PIVKA-II (protein induced by vitamin K absence or antagonism–II) in a dose-dependent manner, implicating that MK-7 improves vitamin K-status in liver, bone, and vasculature (24, 231). In osteoporotic patients, vitamin K2 resulted in elevated levels of cOC and prevented fractures when compared with placebo-treated osteoporotic patients (232). Moreover, both MK-4 and MK-7 supplementation resulted in an increase of cOC and a decrease of ucOC and improved BMD (229, 233–238).

Besides its beneficial effects on bone health, high intake of MK-7 successfully blocked age-related vascular stiffening (239) in post-menopausal women. Moreover, MK-7 was better than placebo at reducing severe aortic calcification and relative risk of coronary heart disease (208, 240). Ongoing clinical trials will evaluate its effectiveness in reducing vascular calcification in patients with coronary artery disease (241). In a cross-sectional study, nutritional long-chain menaquinone intake was associated with decreased coronary calcification in post-menopausal women (240, 242). Moreover, MK-7 improved arterial stiffness and elastic properties of the carotid artery in a healthy postmenopausal woman (20) and improved vitamin K status in dialysis patients by decreasing inactive levels of MGP by daily supplementation (24). In another randomized clinical study, K1 supplementation slowed the progression of CAC in healthy older adults with preexisting CAC, demonstrating the potential efficacy of vitamin K treatment for vascular calcification. Inactive MGP (dp-ucMGP) has been correlated with severity of CKD and is positively associated with amount of vascular calcification (24, 224, 243, 244). MK-7 (MenaQ7) supplementation in patients with CKD3-5 significantly reduced circulating levels of dp-ucMGP (24).

Collectively, these data imply that vitamin K could serve as complementary nutrient to calcium (and vitamin D) to protect from increased risk for vascular calcification thereby allowing more safe treatment of osteoporosis. Vitamin K supplementation in post-menopausal patients appeared beneficial in combination with calcium and vitamin D3 for bone health and vasculature (239). The combination of vitamin K and calcium could reduce risk on post-menopausal bone and simultaneously prevent vascular calcification, thereby aiding the beneficial effects of calcium in bone and preventing the negatively associated vascular effects of supplemental calcium intake.

## Conclusions

To date, calcium supplements are the most commonly used non-prescription drug to treat age-related bone loss. Also, in patients suffering from chronic kidney disease, calcium-based phosphate binders are commonly prescribed. However, the rising concern of side-effects from calcium supplementation illustrates a clinical dilemma: supplementation of calcium—either with or without vitamin D—comes at the price of increased risk of vascular calcification. Clinical studies demonstrate that increased intake of vitamin K could be a promising complementary nutrient in supporting both bone health and protecting vascular calcification. Thereby it can increase safety of current treatments of osteoporosis and provide an escape from the calcium paradox. Future clinical trials should be carried out to confirm the feasibility of such combination.

## Author Contributions

All authors listed have made a substantial, direct and intellectual contribution to the work, and approved it for publication. GW wrote the manuscript. MV wrote the manuscript and was responsible for the final version. LS wrote the manuscript, supervised writing process and was responsible for the final version.

### Conflict of Interest Statement

Nattopharma ASA received an industrial Ph.D. grant from the Norwegian Research Council to conduct research in collaboration with the Maastricht University. GW has been employed as Ph.D. student to work on this project. Nattopharma ASA is a pharmaceutical company with interest in vitamin K2. The remaining authors declare that the research was conducted in the absence of any commercial or financial relationships that could be construed as a potential conflict of interest.
